# 508 Assessing Circulating Phospholipid Vesicles Supporting Coagulation in Major Thermal Injury Patents Receiving Plasma Inclusive Resuscitation

**DOI:** 10.1093/jbcr/iraf019.137

**Published:** 2025-04-01

**Authors:** Samuel DiPasquale, Thomas Orfeo, Meghan Fondakowski, Matthew Gissel, Quinn Chapman, Melissa McLawhorn, Anthony Pusateri, Lauren Moffatt, Jeffrey Shupp, Maria Cristina Bravo

**Affiliations:** University of Vermont; University of Vermont; University of Vermont; University of Vermont; Case Western Reserve University; MedStar Health Research Institute; Naval Medical Research Unit San Antonio; MedStar Health Research Institute; MedStar Washington Hospital Center; University of Vermont

## Abstract

**Introduction:**

In response to major thermal injury stressed or damaged cells release microvesicles (MVs) from their plasma membranes. MVs are membrane bound particles (100-1000 nm diameter) which may present phosphatidylserine (PS) on their exterior surface. PS is a required component of lipid surfaces that support the assembly and function of two major coagulation complexes, the prothrombinase complex and the intrinsic tenase complex. The capacity to support these complexes suggests that elevated levels of circulating MVs may play a role in the progression of burn induced coagulopathy. Studies quantifying PS expressing MVs in thermal injury and the effects of fresh frozen plasma (FFP) administration on these levels are lacking.

**Methods:**

Patients (n=23) with varying burn severity (% TBSA: 40 ± 19, range 19-81%) were enrolled prospectively in an IRB approved study. Blood samples were collected prior to administration of the first transfusion unit (BD1: time from injury 383 ± 249 min) and following transfusion of that unit (BD2: 25 ± 11 min post BD1), platelet poor plasma was prepared from each blood collection. Plasma samples from patients and administered FFP units were thawed, and extracellular vesicle populations were isolated via gel filtration. Total vesicles and their size distribution quantified using a pressurized nanopore system. Gel filtration isolates were mixed with purified FVa, FXa and prothrombin, and thrombin generation was monitored with a chromogenic substrate. Thrombin generation rates by isolates were converted to PS equivalents by reference to a standard curve constructed by combining the same proteins with varying concentrations of PS-bearing vesicles. Data are presented as mean ± SD; paired t-tests were used to compare findings across visits and unpaired across patient samples and FFP units.

**Results:**

PS levels at BD1 (733 ± 560 nM) were not significantly different from those at BD2 (556 ± 274 nM, p>0.3). Mean MV concentration slightly decreased (p=0.06) from pre-transfusion (BD1 = 1.6 ± 1.8 *10^11 particles/mL) to post-transfusion (BD2 = 1.1 ± 1.1 *10^11 particles/mL) but was not significant. There was not a correlation between MVs and PS expression. Concentrations of PS equivalents in FFP units (95 ± 78 nM, n=20) were significantly lower than those seen in BD1 and BD2 (both p< 0.0001).

**Conclusions:**

Functional PS levels were elevated ~ 7-fold in burn patient plasmas compared to plasmas from healthy donors. A single transfusion unit of FFP did not alter the level of PS expressing MVs

**Applicability of Research to Practice:**

The presence of elevated levels of PS expressing vesicles capable of supporting the assembly and function of procoagulant complexes may contribute to dysregulated coagulation in thermal injury and suggests that blockade of these binding sites could have some therapeutic value.

**Funding for the Study:**

USAMRAAA – BA190086

